# Rapeseed Stand Count Estimation at Leaf Development Stages With UAV Imagery and Convolutional Neural Networks

**DOI:** 10.3389/fpls.2020.00617

**Published:** 2020-06-10

**Authors:** Jian Zhang, Biquan Zhao, Chenghai Yang, Yeyin Shi, Qingxi Liao, Guangsheng Zhou, Chufeng Wang, Tianjin Xie, Zhao Jiang, Dongyan Zhang, Wanneng Yang, Chenglong Huang, Jing Xie

**Affiliations:** ^1^Macro Agriculture Research Institute, College of Resource and Environment, Huazhong Agricultural University, Wuhan, China; ^2^Key Laboratory of Arable Land Conservation (Middle and Lower Reaches of Yangtze River), Ministry of Agriculture, Wuhan, China; ^3^Aerial Application Technology Research Unit, USDA-Agricultural Research Service, College Station, TX, United States; ^4^Department of Biological Systems Engineering, University of Nebraska–Lincoln, Lincoln, NE, United States; ^5^College of Engineering, Huazhong Agricultural University, Wuhan, China; ^6^College of Plant Science and Technology, Huazhong Agricultural University, Wuhan, China; ^7^Anhui Engineering Laboratory of Agro-Ecological Big Data, Anhui University, Hefei, China; ^8^College of Science, Huazhong Agricultural University, Wuhan, China

**Keywords:** stand counting, field-based phenotyping, optimal observation timing, convolutional neural network, precision agriculture

## Abstract

Rapeseed is an important oil crop in China. Timely estimation of rapeseed stand count at early growth stages provides useful information for precision fertilization, irrigation, and yield prediction. Based on the nature of rapeseed, the number of tillering leaves is strongly related to its growth stages. However, no field study has been reported on estimating rapeseed stand count by the number of leaves recognized with convolutional neural networks (CNNs) in unmanned aerial vehicle (UAV) imagery. The objectives of this study were to provide a case for rapeseed stand counting with reference to the existing knowledge of the number of leaves per plant and to determine the optimal timing for counting after rapeseed emergence at leaf development stages with one to seven leaves. A CNN model was developed to recognize leaves in UAV-based imagery, and rapeseed stand count was estimated with the number of recognized leaves. The performance of leaf detection was compared using sample sizes of 16, 24, 32, 40, and 48 pixels. Leaf overcounting occurred when a leaf was much bigger than others as this bigger leaf was recognized as several smaller leaves. Results showed CNN-based leaf count achieved the best performance at the four- to six-leaf stage with F-scores greater than 90% after calibration with overcounting rate. On average, 806 out of 812 plants were correctly estimated on 53 days after planting (DAP) at the four- to six-leaf stage, which was considered as the optimal observation timing. For the 32-pixel patch size, root mean square error (RMSE) was 9 plants with relative RMSE (rRMSE) of 2.22% on 53 DAP, while the mean RMSE was 12 with mean rRMSE of 2.89% for all patch sizes. A sample size of 32 pixels was suggested to be optimal accounting for balancing performance and efficiency. The results of this study confirmed that it was feasible to estimate rapeseed stand count in field automatically, rapidly, and accurately. This study provided a special perspective in phenotyping and cultivation management for estimating seedling count for crops that have recognizable leaves at their early growth stage, such as soybean and potato.

## Introduction

Next to soybean and oil palm, rapeseed (*Brassica napus* L.) is the third largest oil crop worldwide (Wang et al., 2010; Berrocoso et al., 2015; Bouchet et al., 2016). Statistical data from the Food and Agriculture Organization of the United Nations have shown that the world production of rapeseed in 2016 was more than 68 million tons, mainly from Canada (19.5 million tons), China (13.1 million tons), and India (6.8 million tons)^1^. Increasing rapeseed yield is a major focus for rapeseed researchers and cultivators (Godfray et al., 2010). The crop stand count at early growth stages is one of the most important parameters for the prediction of yield, density, and growth status (Jin et al., 2017; Liu S. et al., 2017; Zhao et al., 2018). Rapeseed leaf development in early growth stages includes cotyledons completely unfolded, first leaf unfolded, two leaves unfolded, three leaves unfolded, until nine, or more leaves unfolded (Weber and Bleiholder, 1990; Lancashire et al., 1991). Overlapping is intense throughout the entire leaf development stage. Moreover, small and irregular spacing makes rapeseed seedlings clustered. As a result, it is hard to detect and count each individual rapeseed seedling (Zhao et al., 2018). The traditional way of counting rapeseed seedlings at early growth stages is based on ground-level investigation which is labor-intensive and time-consuming (Jin et al., 2017; Liu T. et al., 2017; Naito et al., 2017). Since rapeseed seedlings are in small plant size, irregular spacing and complex overlapping at their early growth stages, timing of rapeseed seedling counting by field investigation depends on empiricism. Accordingly, the obtained records and data are subjective (Bucksch et al., 2014; Deng et al., 2018). The most serious problem is that ground-level investigation is destructive to the field crops (Jin et al., 2017; Liu S. et al., 2017; Deng et al., 2018). In addition, manual investigation brings more external factors into the plant growth environment, resulting in artificial error (Zhao et al., 2018). Therefore, an objective, precise, and automated rapeseed stand counting method will benefit researchers and producers (Araus and Cairns, 2014).

In recent years, plant scientists worldwide have shown great interest in phenotyping since this technology will bring a brand new perspective for agricultural planting and breeding (Furbank and Tester, 2011; Yang et al., 2014; Naito et al., 2017). Phenotyping provides a new tool to reveal phenotype traits determined by environmental and genetic factors (White et al., 2012; McCouch et al., 2013; Ghanem et al., 2015) and to estimate the growth status of plants and crops (Sadras et al., 2013; Maimaitijiang et al., 2017; Yang et al., 2017). Remote sensing technology provides an efficient means for crop phenotype data collection (Verger et al., 2014; Shi et al., 2016; Yu et al., 2016; Wendel and Underwood, 2017), which can record phenotyping traits, such as plant height, canopy temperature, architecture, stress, and color (Walter et al., 2012; Rahaman et al., 2015; Mir et al., 2019). In particular, UAVs draw much attention due to their unique advantages, such as noninvasive observation at low altitude, high resolution, frequent data collection, and deployment flexibility (Zhang and Kovacs, 2012; Ballesteros et al., 2014; Huang et al., 2016). Accordingly, UAVs are used as a platform to collect data and estimate vegetation growth parameters including biomass (Bendig et al., 2014), leaf area index (Córcoles et al., 2013), height (Van Iersel et al., 2018), yield (Geipel et al., 2014), canopy cover, and structure (Cunliffe et al., 2016). Overall, as a powerful and reliable platform, UAVs have shown their advantages to be used to collect crop data for phenotyping.

In studies of crop stand counting, Gnädinger and Schmidhalter (2017) found maize plant numbers had a strong correlation (*R*^2^ = 0.89) with the enhanced color digital counts using UAV imagery. Jin et al. (2017) extracted 13 object features containing color and texture from UAV images and further employed SVM for wheat classification, counting, and density estimation. Their results indicated that wheat density can be estimated when wheat plants had one to two leaves (Jin et al., 2017). Besides, field imagery for crop stand counting has been applied in some other crops such as corn (Shi et al., 2013; Varela et al., 2018), potato (Zheng et al., 2016; Sankaran et al., 2017), and cotton (Chen et al., 2018) with various remote sensing platforms. However, in most previous studies of crop stand counting, data were derived only from one observation at a certain day during the growth stages, and to our knowledge, there has been little research with data from multiple observations for rapeseed.

Object identification, classification, and counting are major tasks in image analysis (Davies, 2009; Blaschke, 2010; Ma et al., 2017; Zanotta et al., 2018). Several approaches have been developed for fast image processing and classification (Schowengerdt, 2012), including SVM, RF. These approaches are developed from using individual spectral, spatial, or textural information to integrating all the information (Linker et al., 2012). Gnädinger and Schmidhalter (2017) used regression analysis to find the correlation between maize plant number and green digital pixel counts based on spectral information. Jin et al. (2017) employed SVM model to classify wheat and to estimate seedling count and density by using spectral and textural features. These approaches can be used for counting trees (Gomes et al., 2018), fruits (Qureshi et al., 2017), and flowers based on satellite remote sensing imagery or UGV/UAV remote sensing imagery. Nevertheless, these regular non-automatic approaches required manual extraction of distinct features, which was time-consuming for a multi-observation study.

Convolutional neural networks have drawn wide attention due to their automated processing and good performance in image analysis (Lee et al., 2017; Pound et al., 2017; Sindagi and Patel, 2017). The CNN application for object detection and counting has been reported in previous studies, such as crowd detection and counting in public events (Sheng et al., 2016; Zhang et al., 2015, 2016) and animal detection and counting in the wild (Arteta et al., 2016). CNN approach is also reported to be applied for classification, detection, and counting for seeds, fruits, flowers, crops, and leaves in agriculture (Grinblat et al., 2016; Ramos et al., 2017). Pound et al. (2017) showed that CNNs were effective to distinguish, identify, and count wheat plants and their ears in glasshouse condition. Madec et al. (2019) used faster region-based CNN (Faster-RCNN) to detect and count wheat ears in a field and estimated the density of ears *via* high-resolution RGB imagery captured by a camera fixed on a boom. Besides, there are some studies using CNN for tree detection and counting. Li et al. (2016) employed a CNN algorithm to detect and estimate oil palm trees from four-band satellite images with 0.6 m spatial resolution, and they reported that more than 96% of trees were correctly detected. Csillik et al. (2018) used a customized CNN model for citrus tree detection and counting with overall accuracy greater than 95%. These studies demonstrated that CNN could be applied to remote sensing imagery captured from both satellites and UAVs. Crop stand counting using CNN has not been widely reported, especially for rapeseed stand counting. Ribera et al. (2017) reported a CNN model to estimate the number of sorghum plants by UAV imagery with a best mean absolute percentage error of 6.7%, which provided a solution for large field plant counting research using CNN. Using CNN in a field or in the wild is of more practical significance (Simonyan and Zisserman, 2014).

However, the situation of crops grown in the field was complex. Specifically, rapeseed seedlings grown in the field were in irregular spacing, complex overlapping, and different plant sizes, which made its counting difficult. Nevertheless, one of rapeseed phenotyping traits that UAV imagery collected was its leaf canopy. During the early growth stages of leaf development, rapeseed leaves play an important role reflecting their growth status (Weber and Bleiholder, 1990; Lancashire et al., 1991). Therefore, it is feasible to recognize individual rapeseed leaves with CNN and perform stand counting with reference to the existing knowledge of the number of rapeseed leaves per plant. The objectives of this study were to (1) recognize and count the individual rapeseed canopy leaves through UAV imagery with CNN, (2) establish and examine the models identifying the number of leaves per rapeseed seedling, and (3) propose an optimal timing to estimate rapeseed stand count.

## Materials and Methods

### Study Area and Experimental Design

**FIGURE 1:**
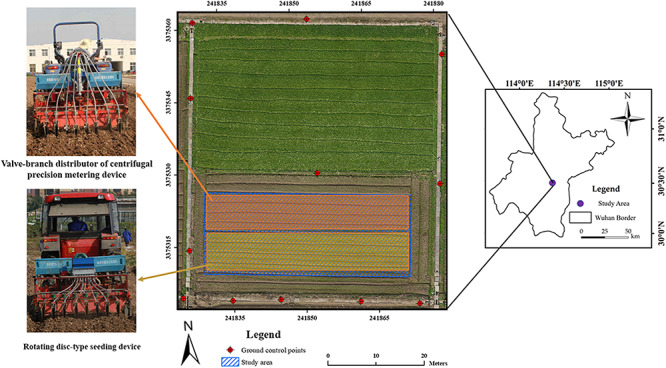
Study area and ground control point (GCP) distribution.

Figure 1 shows the study area and GCPs. The study area with center coordinates (30°28′57.11″N, 114°18′39.45″E) located near Huazhong Agricultural University in Wuhan, China. It covered an area about 50 m × 30 m with an average elevation of 27 m. In the field, a rapeseed cultivar named Huayouza 62 (*B. napus* L.) was sown with two planters including a valve-branch distributor-based centrifugal precision metering device and a rotating disk-type seeding device on November 4, 2017 (Figure 1). According to the experimental design, rapeseed was seeded by the two devices in eight rows simultaneously with 20 cm row spacing at a seeding rate of 5.5 kg/ha. No weed control management was implemented after sowing. There were 12 GCPs permanently arranged surrounding the study area. Reach RS+ (Emlid, United States) was used to collect GPS information.

### Image Acquisition System

The image acquisition system was composed of a DJI rotocopter Matrice 600 UAV (DJI, Shenzhen, China) and a Nikon D800 camera (Nikon, Japan) in this study. The UAV could hover for 35 min without a payload. In this study, each mission took about 13 min with a payload of 2 kg. A GPS module was integrated into the UAV, which was tested with a horizontal accuracy of 0.5 m and a vertical accuracy of 1.5 m. The Nikon D800 camera fitted with a Nikon 50.0 mm f/1.4D lens was mounted on the UAV to collect nadir RGB images of the rapeseed field during flights. The complementary metal oxide semiconductor (CMOS) sensor of the camera had a size of 35.9 mm × 24.0 mm, capturing images with 7,360 × 4,912 pixels. The camera was also integrated with a GPS device to geotag the images and a wireless control to trigger the camera capturing imagery every 1.0 s automatically. An SD memory card was used to store JPEG images with a 24-bit format.

Each flight mission followed the same camera configuration during the whole rapeseed leaf growth stage. The frontal and side overlaps of the flight path were 80.0 and 70.0%, respectively. Two perpendicular flight paths were conducted to cover and image the rapeseed canopy. In this study, the UAV was flown at a ground speed of 3 m/s and at a height of approximately 30 m above ground level, and spatial resolution of image was about 0.18 cm.

### Unmanned Aerial Vehicle Data Collection and Preprocessing

Image collection was scheduled from November 17, 2017, the 14th DAP, to January 12, 2018, with an interval of 7 days. This period covered the whole rapeseed leaf development stage (Weber and Bleiholder, 1990; Lancashire et al., 1991). Some adjustments were made because of rainy or heavy windy weather. As a result, data collection started on November 17, 2017, and ended on January 10, 2018, with specific dates on 14, 23, 32, 39, 46, 53, 58, and 68 DAP from 12:30 p.m. to 2:00 p.m. The one- to three-leaf stage was before 40 DAP and the three- to four-leaf stage lasted from 40 to 50 DAP. The four- to six-leaf stage ranged from 50 to 60 DAP, and the seven-leaf stage and beyond was after 60 DAP.

**FIGURE 2:**
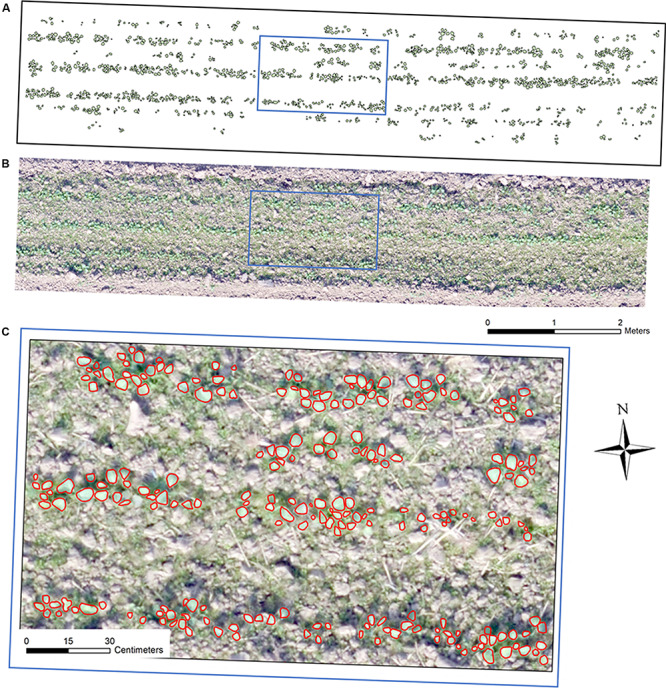
Ortho-mosaic RGB image of a sample plot with a size of 9.5 m × 2 m: **(A)** manual interpreted and annotated rapeseed leaves, **(B)** ortho-mosaic RGB plot image, **(C)** RGB image overlapping with annotated rapeseed leaves.

Free software Capture NX-D 1.2.1 from Nikon (Nikon, Japan) was utilized to correct geometric distortion for the UAV captured individual images. Secondly, an ortho-mosaic image for each collection was generated using Pix4Dmapper software (Pix4D, Switzerland). In this step, the 12 GCPs were added for image mosaicking. This study used eight sample plots (four plots per seeding device) for analysis, which were subsets from each ortho-mosaic image by ArcMap 10.3 (ESRI, United States). Each plot was in the size of 9.5 m × 2 m (Figure 2B).

These eight sample plots were divided into an image training dataset containing six sample plots and a test dataset consisting of two sample plots during the whole processing and analysis. Since manual counting in the field could possibly change the real field condition, making the data unreliable for multiple observations, this study manually interpreted and annotated the rapeseed leaves over the eight sample plots for each observation date using ArcMap 10.3 (an illustration in Figure 2), which was mainly described in the next section. This study also used image-based manual rapeseed seedling count as the ground truth reference. Data of 14 and 23 DAP were not used because rapeseed seedlings were too small to distinguish in imagery. Therefore, there were six remaining observation dates over the eight study plots (six for training and two for testing) in this study.

All the processing and analysis were executed on a computer with an Intel (R) Core (TM) i7-6800K CPU and one NVIDIA GeForce GTX 1060 6GB GPU, and the memory of the computer was 32 GB.

### Image Processing and Data Analysis

#### Rapeseed Leaf Recognition Based on a Convolutional Neural Network

Convolutional neural networks are the most popular machine learning algorithms applied to various computer vision tasks, such as numeral recognition, face recognition, and handwriting recognition. Some software packages such as Python and Matlab provide a convenient environment for CNN modeling, and there are some open-source CNN codes online. However, it is still hard for a nonprofessional machine learning researcher to implement the entire flowchart of CNN modeling, including CNN software operation environment configuration, codes modification, as well as parameter adjustment. In addition, such CNN software is not suitable for processing geospatial information and geoinformation analysis. Thus, this study employed the easy-to-use image analysis software eCognition Developer 9.3 (Trimble, United States), which contains a CNN module (Trimble, 2018). The module can be used to recognize objects in images based on the Google TensorFlow^TM^ library-create (Csillik et al., 2018; Trimble, 2018).

This approach was a patch-based CNN algorithm according to the categorization reviewed by Sindagi and Patel (2017). It was convenient and interactive when researchers use CNN for image analysis in this software. The operation in this software was mainly composed of four steps: (1) to generate labeled sample patches; (2) to create a CNN; (3) to train a CNN; and (4) to apply a CNN (Trimble, 2018). In this study, the labeled sample patches were cropped from the training sample plot images according to three classes including rapeseed leaves, weeds, and bare soil.

To label the three classes, the rapeseed leaves from the eight sample plots for each observation date were manually interpreted and annotated using ArcMap 10.3, as shown in Figure 2A. The labeled leaves were used as a reference, representing the ground truth leaves. The overlapping of rapeseed leaves was unavoidable, and each recognized canopy rapeseed leaf was outlined. Furthermore, the weeds increased with time since there was no weed control management in the field to maintain the original ecological scene. As DAP increased, the rapeseed leaves and weeds were much distinguishable by size, color, texture, and pattern in high-resolution UAV images. Therefore, after interpreting and annotating the rapeseed leaves, we used ExG-ExR, a color vegetation index whose ability for green pixel identification was confirmed (Meyer and Neto, 2008; Zhao et al., 2018) to label the remaining green pixels as weeds by eCognition Developer. The pixels that were not in green were labeled as bare soil through normalized green minus red difference index (NGRDI). The pixels whose values of NGRDI less than 0 were classified as bare soil. In this study, the input data of eCognition Developer include RGB imagery in TIFF format exported from ArcMap, the manual-annotated rapeseed leaf polygon shapefile exported from ArcMap.

**FIGURE 3:**
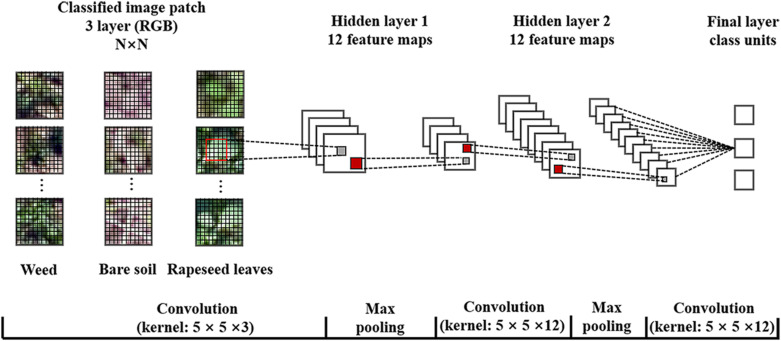
Structure and parameters of the convolutional neural network (CNN) model used in this study. RGB, red-green-blue.

The parameters during the operation included sample patch size, number of hidden layers, kernel size, number of feature maps, max pooling, and learning rate. According to the rapeseed leaf development stages from one leaf to more than seven leaves during the investigation period, this study generated five sizes of sampling patches for CNN training, which included 16 × 16, 24 × 24, 32 × 32, 40 × 40, and 48 × 48. In addition, the default number of feature maps was 12, while the learning rate was at 0.0005 in the software. Based on the five patch sizes, the optimal kernel size was 5 × 5 after trial-and-error processing. Therefore, this study structured an initial CNN model as shown in Figure 3, containing two hidden layers, two max pooling layers, and one fully connected layer. A previous study using a similar CNN model showed great performance for oil palm tree detection and counting with satellite images (Li et al., 2016). We generated 20,000 sample patches of rapeseed leaves, 10,000 weed patches, and 10,000 bare soil patches from the training dataset in each observation date for five sample patches totally.

**FIGURE 4:**
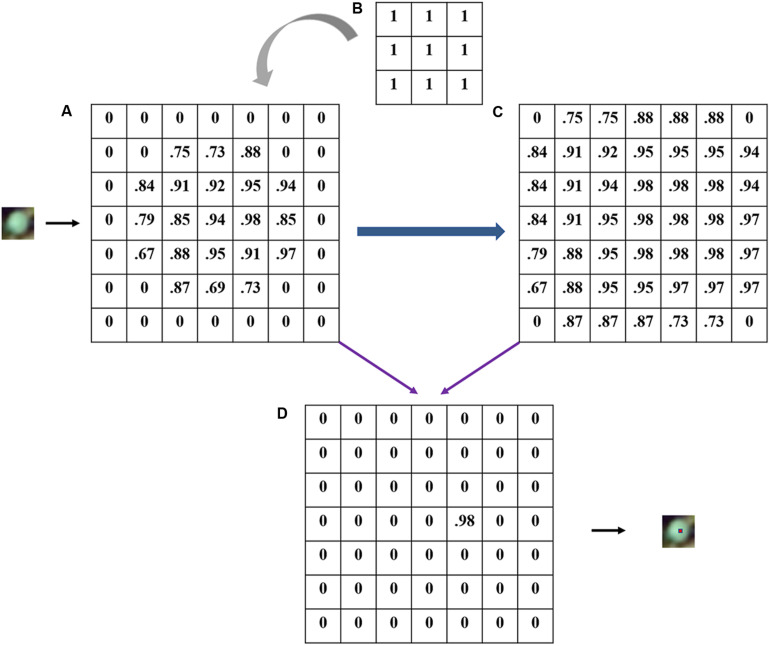
An illustration of local maximal possibility detection with 3 × 3 filter (actual 5 × 5 in the study): **(A)** possibility heat map; **(B)** a 3 × 3 dilation filter; **(C)** the maximal layer after dilating; **(D)** locating target.

The number of recognized rapeseed leaves was counted after the dilate operation in the outputted heat map, according to the number of local maximal points. A heat map with each pixel value ranging from 0 to 1 was output by the CNN model, representing the possibility of the target class for rapeseed leaves. High pixel values close to 1 in the heat map indicated a high possibility of rapeseed leaves, while values close to 0 indicated a low possibility (Csillik et al., 2018; Trimble, 2018). Based on the pixel values, local maximal pixels whose threshold ranged from 0.5 to 1 (step on 0.01) were iteratively searched to locate a rapeseed leaf through the dilate operation in eCognition as the locations of rapeseed leaves were expected to coincide with local maximal and high values in the heat map (Figure 4).

In this study, the size of the dilation filter was defined as 5 × 5, which was the same as the kernel size in CNN. Afterward, a maximal layer was generated, reflecting the maximum value in the matrix of 25 pixels in the heat map (Figure 4C). Accordingly, when a pixel in the maximal layer has the same value with the pixel in the same location of the heat map, this pixel inherited the value. Otherwise, the pixel was valued 0 (Figure 4D). Each located point was considered to represent a CNN-detected rapeseed leaf. Thus, the number of local maximal points was considered as the number of recognized rapeseed leaves.

**FIGURE 5:**
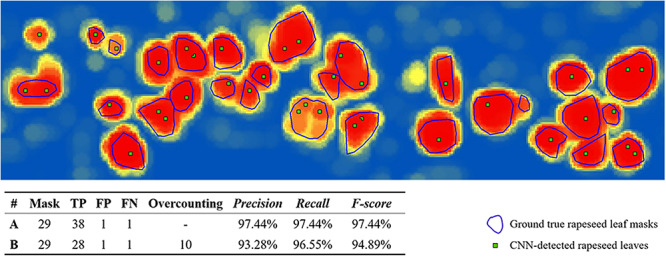
Illustration of overcounting statistics. CNN, convolutional neural network; FN, false negative; FP, false positive; TP, true positive. A was calculated without overcounting rate, B was calculated with overcounting rate.

Overcounting occurred when the number of CNN-detected rapeseed leaves sometimes was larger than the actual number of rapeseed leaves, as a big leaf might correspond to several located maximum points (Figure 5). In Figure 4, if a pixel far away from the pixel of 0.98 also had a value of 0.98, two targets might be located in Figure 4D. Merging the adjacent points with a tolerance was a useful means for tree canopy recognition and counting (Li et al., 2016), but this method cannot be applied to rapeseed leaf counting in this study since the boundary of rapeseed leaves was less distinct than that of a tree canopy. Moreover, the sizes of rapeseed leaves were also different. Therefore, it was difficult to determine a precise distance for merging rapeseed leaf located points. Inspired by the method of using ground truth masks for assessing the accuracy of the estimated plant centers (Chen et al., 2017), we used the manual interpreted ground truth rapeseed leaf outlines as masks to record and analyze the overcounting rate (*R*_oc_) of the local maximum points for each investigation (Figure 5).

The illustration in Figure 5 was a subset of CNN recognition results on 53 DAP with the 32-pixel patch size and a local max value of 0.65. The blue lines showed rapeseed leaf outline masks, and each mask represented a ground truth rapeseed leaf. The green points illustrated the CNN-detected rapeseed leaves.

As mentioned, the sizes of leaves influenced the number of located maximum points representing the CNN-detected rapeseed leaves, and DAP was a significant factor of the leaf size. Thus, it could be assumed that in our study duration, the *R*_oc_ might be related to DAP. For CNN detection and counting, *R*_oc_ was calculated by:

(1) $$R_{o\,c}=\frac{\sum_{i=1}^{M}\left(C_{i}-1\right)}{N}\times 100\%,\left(\text{when},C_{\text{i}}>1\right)$$

where *N* is the number of CNN-detected rapeseed leaves, *M* is the number of ground truth rapeseed leaf masks, *C*_i_ is the number of located maximum points inside a ground truth rapeseed leaf mask for mask *i*, when mask *i* has more than one point.

*Precision*, *Recall*, and *F-score* were used in this study to evaluate leaf detection results (Xiong et al., 2017; Zhao et al., 2018). *Precision* and *Recall* are defined by true positive (TP), false positive (FP), and false negative (FN):

(2) $$P\,r\,e\,c\,i\,s\,i\,o\,n=\,\frac{\text{TP}}{\text{TP}+\text{FP}}$$

(3) $$R\,e\,c\,a\,l\,l=\frac{\text{TP}}{\text{TP}+\text{FN}}$$

Combined with the situation of overcounting, the following equations can be inferred by Eqs. (1), (2), and (3) in this study:

(4) $$N=\mathrm{TP}+\text{FP}+\sum_{i=1}^{M}\left(C_{i}-1\right)$$

(5) M=TP+FN

The unique recognized local maximum point that is inside a ground truth mask is considered as a TP. Accordingly, TP here is the accurate number of CNN-detected rapeseed leaves. If a local maximum point is outside a mask, then this maximum point is considered as a FP. A mask is identified as a FN if there is no point recognized inside (Figure 5). *F-score* was used as the final exponent to evaluate the CNN-detected rapeseed leaf recognition accuracy. *Precision*, *Recall*, and *F-score* in this study can be expressed as follows:

(6) $$P\,r\,e\,c\,i\,s\,i\,o\,n=\,\frac{\text{TP}}{\left(1-R_{o\,c}\right)\times N}$$

(7) $$R\,e\,c\,a\,l\,l=\frac{\text{TP}}{M}$$

(8) $$F-s\,c\,o\,r\,e=\frac{2\times P\,r\,e\,c\,i\,s\,i\,o\,n\times R\,e\,c\,a\,l\,l}{\left(P\,r\,e\,c\,i\,s\,i\,o\,n+R\,e\,c\,a\,l\,l\right)}\,$$

#### Leave-One-Out Crossing Validation Regression Modeling for Rapeseed Seedling Counting

A strong relationship between the rapeseed seedling counting and the number of unfold leaves was expected because the identification of rapeseed at early growth stages was based on the number of unfolded leaves (for example, the one- to three-leaf stage and four- to six-leaf stage). In this study, the models that counting rapeseed seedlings through the number of manual-interpreted rapeseed leaves were first established and verified, which were considered as the reference describing the number of leaves per rapeseed seedling plant. Then the number of CNN-recognized rapeseed leaves was applied to these models to evaluate the accuracy of rapeseed stand count estimation at different growth stages.

The LOOCV regression modeling method was used to establish the models of seedling stand counting. LOOCV was effective in the case of small sample size. It was a special case of K-fold crossing validation, when K was equal to the number of samples. One sample was excluded for validation, and the rest samples were used for training. The same operation was repeated for K times so that each sample could be used for validation so that the results were unbiased. The LOOCV regression modeling was conducted by Python Spyder in Anaconda3 (64-bit) (Anaconda Software Distribution, 2016. Computer software).

In this study, there were six training sample plots for each investigation. Thus, K was 6, and LOOCV regression modeling was repeated for six iterations. The optimal rapeseed seedling model parameters of the corresponding DAP were further obtained by calculating an average value of the iteration results. Mean absolute error, RMSE, and coefficient of determination ($R_{\text{LOOC}}^{2}$) were used to verify these models. They were calculated as follows:

(9) $$\text{RMSE}=\,\sqrt{\frac{\sum_{i=1}^{n}{\left(y_{i}-{\hat{y}}_{i}\right)}^{2}}{n}}$$

(10) $$\text{MAE}=\,\frac{1}{n}\,\sum_{i=1}^{n}\left|y_{i}-{\hat{y}}_{i}\right|$$

(11) $$R_{L\,O\,O\,C}^{2}=1-\frac{\sum_{i=1}^{n}{\left(y_{i}-{\hat{y}}_{i}\right)}^{2}}{\sum_{i=1}^{n}{\left(y_{i}-\bar{y}\right)}^{2}}\;$$

where *n* is the number of sample plots, *y*_i_ is the investigated ground truth rapeseed seedling stand count for sample *i*, ${\hat{y}}_{\text{i}}$ is the model-predicted rapeseed seedling stand count for sample *i*, $\bar{y}\,$ is the average value of the investigated ground truth rapeseed seedling stand count for all samples in each observation date.

#### Performance Evaluation of Counting Seedlings

The number of CNN-recognized rapeseed leaves was used to evaluate the eventual performance of counting seedlings in this study. The number of CNN-recognized rapeseed leaves corresponding to the best value of *F-score* was applied to the models. Relative RMSE (rRMSE) was calculated as follows:

(12) $$\text{rRMSE}=\,\frac{\text{RMSE}}{\bar{y}}\times 100\%$$

where $\bar{y}$ is the average value of the investigated ground truth rapeseed seedling stand count for all samples in each observation date.

## Results

### Counting Rapeseed Leaves Recognized by Convolutional Neural Networks

**FIGURE 6:**
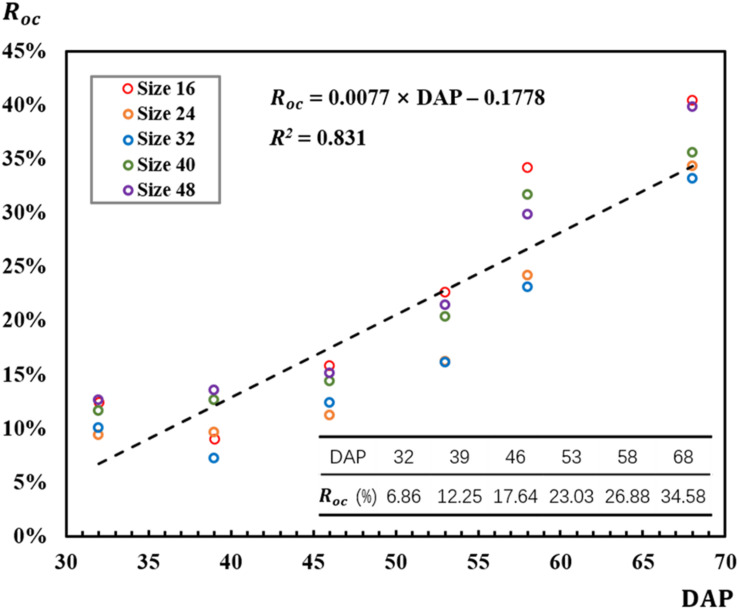
Correlation between overcounting rate and days after planting (DAP).

The strong correlation (*R*^2^ = 0.831) between *R*_oc_ and DAP conformed the impact of DAP on *R*_oc_ that *R*_oc_ increased as DAP advanced during the observed rapeseed leaf development stages (Figure 6). The bigger leaves caused the more detected local maximal points for a leaf, thus resulting in the overcounting situation. As shown in Figure 6, *R*_oc_ ranged from 7% on 39 DAP for the 32-pixel patch to 40% on 68 DAP for the 16-pixel patch. According to the correlation in Figure 6, *R*_oc_ was estimated to be 6.86% for 32 DAP, 12.25% for 39 DAP, 17.64% for 46 DAP, 23.03% for 53 DAP, 26.88% for 58 DAP, and 34.58% for 68 DAP. In Figure 6, *R*_oc_ for the 32-pixel patch (blue legend) was lower than that for the other four patch sizes in most DAPs.

**FIGURE 7:**
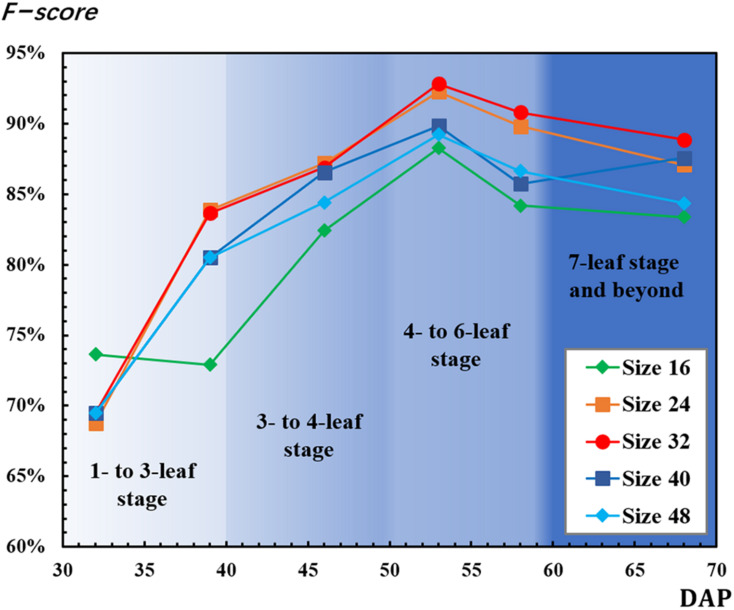
Results of *F-score* for convolutional neural network (CNN)-detected rapeseed leaf recognition and counting.

*F-scores used for* CNN-based recognition accuracy evaluation were calculated by equations (9), (10), and (11). The mean *F-scores* based on local maximal values ranging from 0.5 to 1 were calculated (Figure 7). For most of the patch sizes, *F-scores* increased from 32 to 53 DAP and decreased after 53 DAP. However, *F-scores* decreased from 32 to 39 DAP for the 16-pixel patch and increased from 58 to 68 DAP for the 40-pixel patch. For all patch sizes, *F-scores* had the highest values on 53 DAP. On 53 DAP, the ranking of *F-score* values from highest to lowest was 92.83% for the 32-pixel patch, 92.26% for the 24-pixel patch, 89.84% for the 40-pixel patch, 89.21% for the 48-pixel patch, and 88.26% for the 16-pixel patch.

**FIGURE 8:**
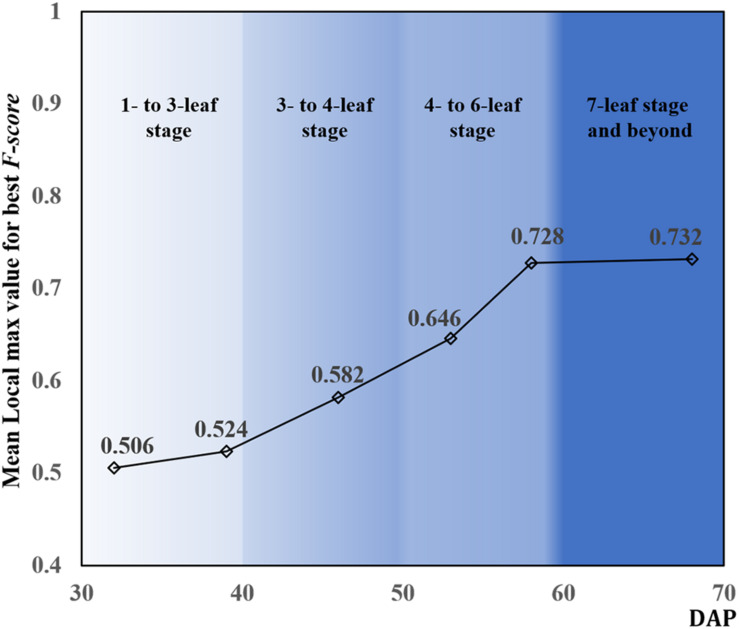
Mean local max values for the best *F-score* according to days after planting (DAP).

Figure 8 shows the mean local max values achieving best *F-score* among the testing data during the whole observed leaf development stage. DAP also influenced the variation of local max values. The local max values increased with DAP and leveled off after 58 DAP. With these local max values, the number of rapeseed leaves recognized by CNN was counted for each observation.

### Revealing the Number of Rapeseed Leaves per Seedling

A strong correlation between the seedling counting and the number of canopy rapeseed leaves was established by using the LOOCV method over data of training sample plots for each observation (Table 1). All sub-models were significant (*p*-value < 0.05) except for the sub-model of the first iteration on 32 DAP exhibiting a *p*-value of 0.058. Afterward, the relationship defining the number of rapeseed leaves per seedling was revealed by the formulas in Table 1 for each observation.

**TABLE 1:** Results of optimal rapeseed seedling counting models for six observation periods.

Period	**Formula**	**$R_{\mathrm{LOOC}}^{2}$**	**MAE**	**RMSE**
32 DAP	y = 0.354x + 14.340	0.775	47	54
39 DAP	y = 0.297x + 65.037	0.897	29	30
46 DAP	y = 0.322x + 19.490	0.984	13	14
53 DAP	y = 0.277x + 41.540	0.926	25	31
58 DAP	y = 0.220x + 72.334	0.886	31	35
68 DAP	y = 0.214x + 46.388	0.806	31	42

*x represents the number of rapeseed canopy leaves. y stands for rapeseed seedling count. MAE and RMSE were rounded to integer. DAP, days after planting; MAE, mean absolute error; RMSE, root mean square error.*

These formulas were verified by the testing data. The ordering of $R_{\mathrm{LOOC}}^{2}$ from highest to lowest was 0.984 on 46 DAP, 0.926 on 53 DAP, 0.897 on 39 DAP, 0.886 on 58 DAP, 0.806 on 68 DAP, and 0.775 on 32 DAP. RMSE ranged from 14 plants to 54 plants, and MAE ranged from 13 plants to 47 plants. Results showed that the relationship revealing the number of rapeseed leaves per seedling obtained the best performance on 46 DAP and satisfactory performance on 53 DAP. As a result of the strong correlation revealing the number of rapeseed leaves per seedling, the approach using precise CNN-recognized leaves counting to estimate the seedling stand count was feasible and expected.

### Performance of Estimating Seedling Stand Count With Convolutional Neural Network-Recognized Leaf Counting

**FIGURE 9:**
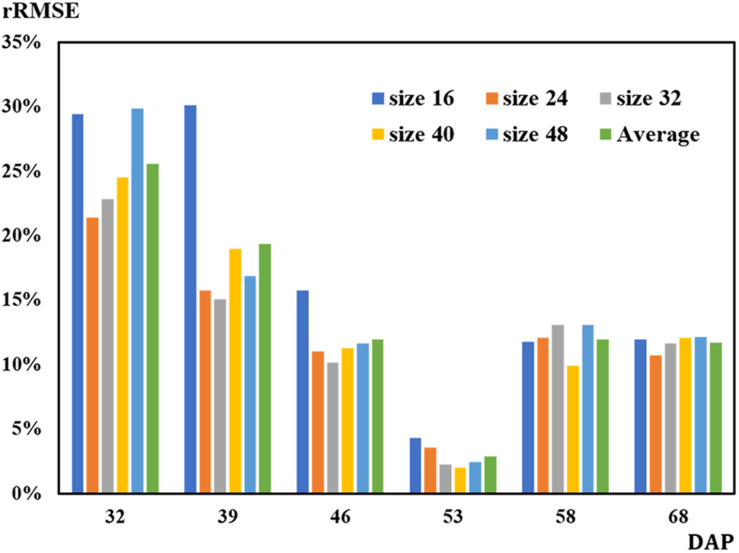
Results of relative root mean square error (rRMSE) for rapeseed stand count estimation.

Convolutional neural networks-recognized leaf counting (see section “Counting Rapeseed Leaves Recognized By Convolutional Neural Networks”) was used to estimate seedling stand count according to the revealed relationship of the number of rapeseed leaves per seedling (Table 1 in section “Revealing the Number of Rapeseed Leaves Per Seedling”). Table 2 gives the results of estimating rapeseed seedling stand count. For all patch sizes, the best mean accuracy was achieved on 53 DAP with 99.26%. On average, 806 out of 812 plants were correctly estimated on 53 DAP at the four- to six-leaf stage. With the 32-pixel patch size, almost all the seedling stand counts were correctly estimated on 53 DAP. Some errors were counteracted mutually as a result of summing up the estimated seedling stand counts from two test sample plots. RMSE and rRMSE are presented in Table 3 and Figure 9, respectively.

**TABLE 2:** Sum of rapeseed stand count estimation from two testing sample plots (unit: plants).

	**Size 16**	**Size 24**	**Size 32**	**Size 40**	**Size 48**	**Average**	**Mean accuracy (%)**	**Ground-truth plants**
32 DAP	593	672	657	640	590	630	75.05	840
39 DAP	567	734	668	640	657	653	83.42	783
46 DAP	686	723	727	712	714	712	89.05	800
53 DAP	801	806	812	804	807	806	99.26	812
58 DAP	835	833	823	854	823	834	88.12	946
68 DAP	803	802	805	805	800	803	96.51	832

*Estimated results were rounded to integer. DAP, days after planting.*

**TABLE 3:** Results of RMSE for rapeseed stand count estimation (unit: plants).

	**Size 16**	**Size 24**	**Size 32**	**Size 40**	**Size 48**	**Average**
32 DAP	124	90	96	103	125	107
39 DAP	118	62	59	74	66	76
46 DAP	63	44	40	45	47	48
53 DAP	17	14	9	8	10	12
58 DAP	56	57	62	47	62	57
68 DAP	50	45	48	50	51	49

*Means the optimal value in the column. Results were rounded to integer. DAP, days after planting; RMSE, root mean square error.*

On 32 DAP, a maximum mean RMSE of 107 plants was observed, while on 53 DAP, a minimum mean RMSE of 12 plants showing the best performance was obtained. On 53 DAP, the rRMSE was 1.99% for the 40-pixel patch size (Figure 9). For the 32-pixel patch size, its RMSE was nine plants with an rRMSE of 2.22%. The ordering of mean RMSE from lowest to highest was 12 on 53 DAP, 48 on 46 DAP, 49 on 68 DAP, 57 on 58 DAP, 76 on 39 DAP, and 107 on 32 DAP, with the best performance on 53 DAP. Similarly, the ordering of rRMSE was 2.89% on 53 DAP, 11.71% on 68 DAP, 11.97% on 46 DAP, 11.97% on 58 DAP, 19.36% on 39 DAP, and 25.59% on 32 DAP.

## Discussion

### Influence of Days After Planting Corresponding to Leaf Development Periods

Huayouza 62 (*B. napus* L.) used in the study is a member of the family Brassicaceae (Weber and Bleiholder, 1990; Lancashire et al., 1991). Based on the characteristics that its leaf development periods were highly related to DAP, the number of canopy leaves was used for seedling stand count modeling and estimation in this study. Chen et al. (2018) found that DAP had a strong correlation with the number of germinated seeds (*R*^2^ = 0.938) and with average plant size (*R*^2^ = 0.936). In our study, results also strongly illustrated that DAP played an important role on the leaf recognition and counting, revealing the relationship between seedling stand count and the number of leaves per seedling, and the eventual estimation of seedling stand count with CNN-recognized leaf counting. Finally, the best performance was achieved on 53 DAP for leaf recognition and counting as well as estimating seedling stand count with the number of CNN-recognized leaf counting (Figures 7, 9 and Tables 2, 3).

This study not only used DAP but also tried to associate it with the leaf development period and to determine the influence of DAP corresponding to the leaf development periods on rapeseed leaf counting (Figures 7, 8). DAP is an essential dimension unit to describe the growth process of crops, and it is easy to comprehend and quantify. Nevertheless, the growth situation based on DAP varies with different regions where crops and plants are cultivated with different treatments, even for the same cultivated variety. Sankaran et al. (2017) reported that it was hard to estimate the number of potato plants after 43 DAP because of the within-row canopy closure, even though the best correlation coefficient (*r* = 0.83) between manual plant counts and image-based counts was achieved on 32 DAP. This study presented a novel approach to understand the growth status of rapeseed seedlings and to count the seedlings in terms of leaf development periods.

In Figure 6, overcounting rate increased with DAP. From another perspective, the increasing overcounting rate was mainly caused by the development of leaves or the increasing size and number of rapeseed seedling canopy leaves. Rapeseed seedlings experience a process from new leaf unfolding to leaves gradually growing with DAP. In this study, rapeseed had more than seven leaves in the last observation period, which caused a serious overlapping. Moreover, the bigger leaf size influenced the recognition. According to the trend and strong correlation between overcounting rate and DAP (*R*^2^ = 0.831), when the number of leaves developed from one to more than seven leaves, overcounting rate might keep increasing.

**FIGURE 10:**
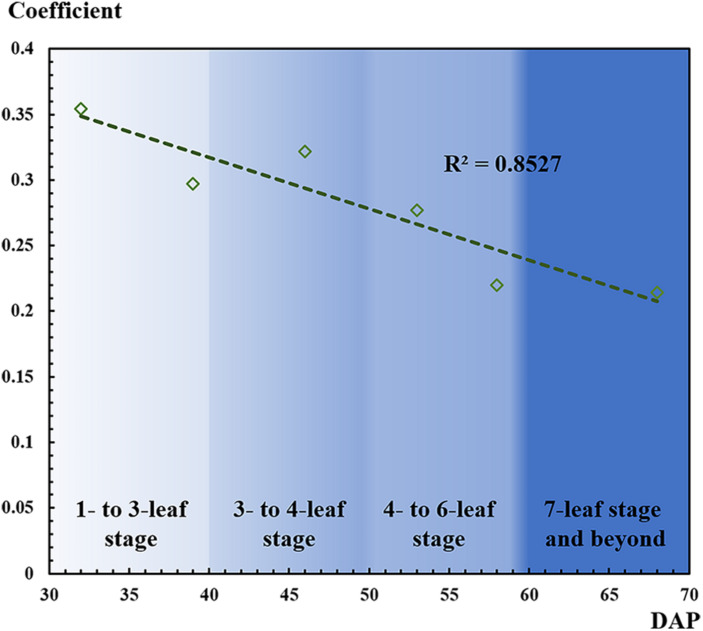
Correlation between coefficient of the seedling counting models and days after planting (DAP).

As mentioned, as the number of leaves increased, leaf overlapping and saturation became more intensified. In Figure 7, F-score increased from the one- to six-leaf stage, and then decreased after that. This peak of F-score was mainly related to the leaf development. When rapeseed seedlings were in the one- to six-leaf stage, the number of unfolded leaves increased dramatically, and the characteristics of leaves became more and more obvious so that the recognition performance improved. After the six-leaf stage, leaf overlapping and saturation resulted in the decreasing recognition accuracy. On the other hand, in Figure 8, the local maximal value for CNN recognition did not increase after the six-leaf stage. Moreover, a strong correlation (*R*^2^ = 0.835) between the coefficient of the seedling counting models (Table 1) and DAP was found (Figure 10).

When a DAP was replaced by its corresponding leaf development stage, the coefficients were approximately the inverse of the number of leaves at the given stage. For example, at the one- to three-leaf stage, the coefficients on 32 and 39 DAP were 0.354 and 0.297, which were approximately the inverse of 3 (1/3 or 0.333). Similarly, at the four- to six-leaf stage, the coefficients on 53 and 58 DAP were 0.277 and 0.220, which were approximately the inverse of 5 (1/5 or 0.200). To some extent, this correlation demonstrated another perspective to understand the counting models through the leaf development stage, but more data are needed to verify the result more scientifically.

Another interesting finding related to the leaf development stage of rapeseed seedlings was that the timing of 53 DAP corresponding to the four- to six-leaf stage was near winter solstice in the Chinese calendar. Winter solstice, a meaningful solar term, is known as a significant time for winter rapeseed planting as well as for other agricultural activities. Many Chinese agronomic researchers and cultivating specialists regard winter solstice as a critical timing for rapeseed characteristic measurement. It is also considered as a mid-growth phase of rapeseed leaf development with about five leaves. In fact, the day of winter solstice was 4 days before 53 DAP. Therefore, the DAP corresponding to the leaf development stage was confirmed to be reliable in this study. Our findings offered a scientific explanation for the agricultural practice in China and should be referential for relevant studies and practices.

### Influence of Parameters in Convolutional Neural Network

#### Influence of Patch Size

It is necessary to define patch sizes according to the specific purposes and the image target for the application of CNN. In this study, sample patches were cropped from the entire labeled UAV image. Moreover, the trained CNN model was applied to a large-scale field image. This study employed five sample patch sizes with 16, 24, 32, 40, and 48 pixels.

These sizes were determined mainly by the image resolution and the growth situation of rapeseed. Madec et al. (2019) assumed that resolution around 0.3 mm could allow to detect and count wheat ears for high-throughput phenotyping based on UAV-captured RGB images using Faster-RCNN. However, their research used imagery captured by a camera fixed on a boom, and their assumption was not verified with UAV images in their work. The imagery used in this study was captured by UAV in the field 30 m above ground level. If the altitude was more than 30 m, the resolution was not enough for recognition. If the UAV imagery was captured below 30 m, it would reduce the efficiency of field plot data collection. Furthermore, the turbulence generated by the UAV made the rapeseed leaves shake, leading to the unfocused targets in the imagery.

Meanwhile, the leaf size of rapeseed was another factor determining the patch size. In this study, an overlarge patch size such as a 128-pixel patch could contain redundant and useless information. On the contrary, an undersized patch would cause underfitting recognition results because the patch would be so small that the features between rapeseed leaves and weeds could not be distinguished. Employing a patch-based CNN algorithm (Sindagi and Patel, 2017) brought about a problem that the patch could not cover an oversized rapeseed leaf, resulting in a larger number of detected objects than the actual number called overcounting in this study (Figure 5). Therefore, the patch size of UAV imagery was limited by its imaging mode and resolution, field status, and the characteristics of objects.

In this study, the areas of the five patches were calculated and matched with the leaf size of rapeseed for each observation. The area of the 48-pixel patch was calculated to be 74.6 cm^2^, and the maximum area of rapeseed leaf obtained on 68 DAP was almost 63 cm^2^. Based on this, a sampling patch cropped with 48 pixels could meet the minimum requirement for covering an entire rapeseed leaf. However, each patch size was used for all the observations, thus the same patch size had a dynamic influence on the counting results as DAP changed. In Figure 7, the 16-pixel patch exhibited the optimal performance on 32 DAP, which was attributed to the fact that the 16-pixel patch could match the leaf size better on 32 DAP than on the other DAPs. On the other DAPs, the 24-pixel patch and the 32-pixel patch matched the leaf size better than the other patch sizes achieving better *F-score* in Figure 7.

**FIGURE 11:**
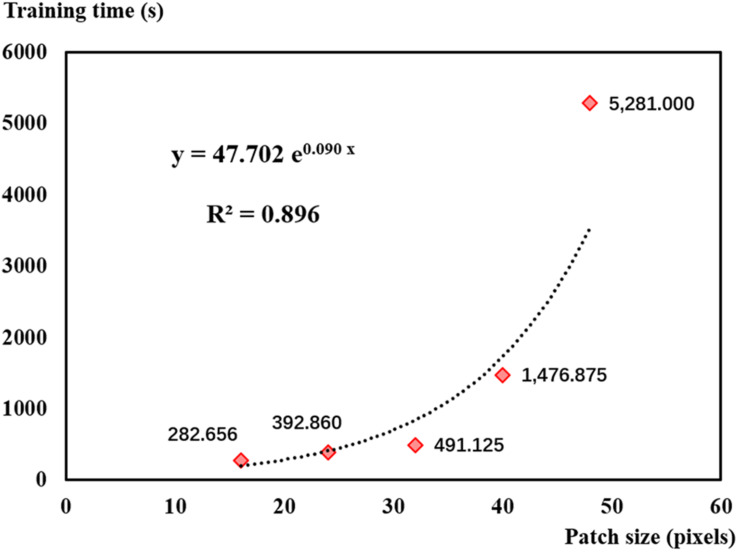
Convolutional neural network training time consumed for five sampling patch sizes.

In addition, patch size significantly affected training time. Figure 11 shows that training time increased exponentially with patch size. Training time was more than 5,200 s (about 90 min) when using the 48-pixel patch. Although using the 16-pixel patch could save time, its results were not desirable in this study. According to Figure 11 and based on the results of leaf recognition and seedling estimation, the 32-pixel patch was selected as the optimal sampling size balancing the performance and efficiency in this study.

#### Influence of Learning Rate

**FIGURE 12:**
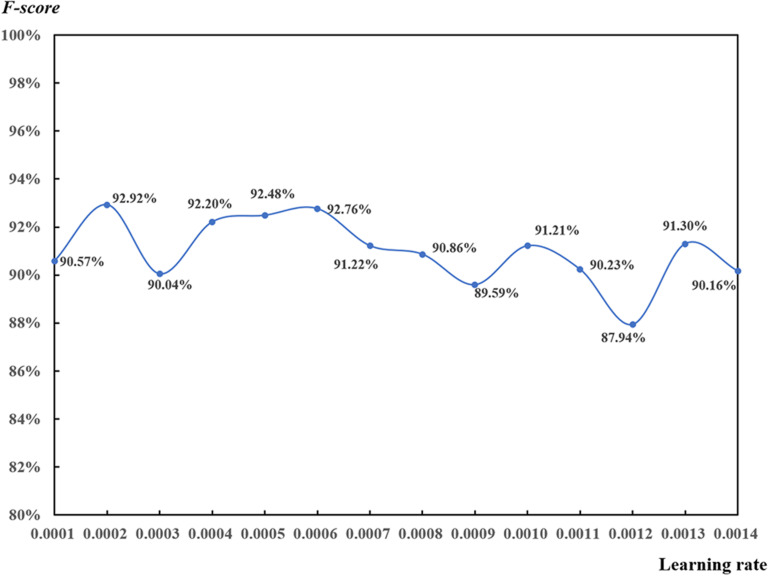
Test of learning rate for the convolutional neural network-based rapeseed leaf recognition on 53 days after planting (32-pixel patch, local max value of 0.65).

Learning rates ranging from 0.0001 to 0.0014 (step on 0.0001) were used to analyze the learning rate function with the 32-pixel patch on 53 DAP based on the local max value of 0.65 (Figure 12). *F-scores were* higher than 90.00% for most of the results. However, it was difficult to determine the relationship between learning rate and *F-score* since there was an irregular fluctuation. In terms of the ranking of *F-scores*, the top four learning rates were 0.0002, 0.0006, 0.0005, and 0.0007 with all their corresponding *F-scores* higher than 92.00%.

Learning rate plays an important role in CNN models (Trimble, 2018). It defines the weights used to adjust the gradient descent optimization. If learning rate is too small, the learning process will be slowed down and may not be close to the optimal settings. If learning rate is too large, the model may not reach the minimum boundary and produce results of null values (Trimble, 2017). Learning rate is considered as a hyper-parameter in machine learning and deep learning, which is mostly set up based on practices and empiricism (Senior et al., 2013). Finally, learning rates ranging from 0.0004 to 0.0006 were suggested in this study.

### Influence of Overcounting Rate

Overcounting is common in object identification and counting tasks, especially in large-scale scenes (Li et al., 2016). It is challenging to use CNN algorithms for object detection and counting in a large-scale scene. Most studies detected flowers, leaves, and crops in the lab because the scenes were well controlled and the results were less influenced by external factors. This study detected rapeseed leaves in a field-based scene. The existing leaf overlapping was one of the main reasons for overcounting. With leaf size increasing, the overlapping became more complex, which caused serious overcounting and inaccurate counting results.

**FIGURE 13:**
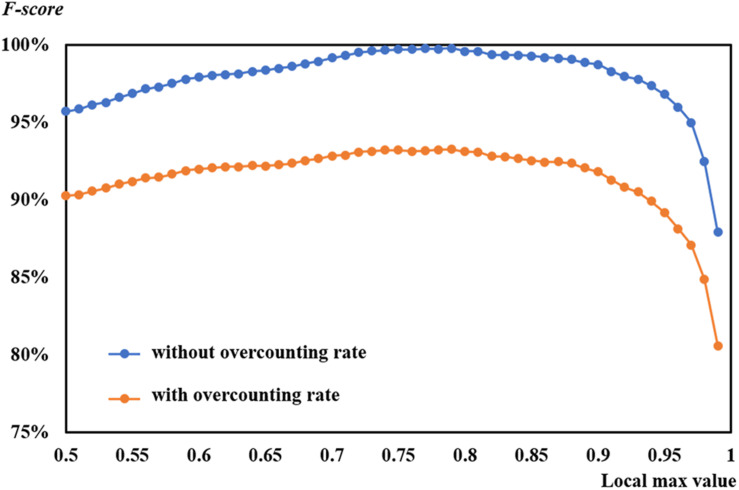
Comparison of *F-scores* with and without overcounting rate at a learning rate of 0.0004 on 53 days after planting.

This study selected and employed different patch sizes, attempting to match the size of leaves and improve model performance. However, the overcounting was still difficult to avoid. Comparison was made between the *F-scores* with overcounting and those without overcounting based on the suggested learning rate of 0.0004 on 53 DAP (Figure 13). Even though two curves of *F-scores* exhibited a similar tendency, *F-scores* without overcounting calibration were higher than those with overcounting calibration. The former almost reached 100%, which was inaccurate.

A graphic example of *F-score* with and without overcounting calibration is shown in Figure 5. As shown in the figure, *precision* without overcounting calibration was greater than that with overcounting calibration, resulting in an illusory high *F-score*. *F-score*s with overcounting calibration more accurately and objectively described the CNN-based leaf recognition performance. When the number of referencing masks was 29, the accurate number of detected rapeseed leaves was 28 using overcounting calibration. Without overcounting calibration, the number of detected rapeseed leaves was 38 (Figure 5). These results demonstrated that overcounting calibration was important to obtain precise rapeseed leaf counting. When overcounting calibration was considered, more reliable and objective rapeseed leaf counting results were achieved as demonstrated by the study.

### Future Work

This study was a specific application of machine learning in agriculture for quantitative analysis of rapeseed seedling stand counting at a field level from UAV images. We aimed to estimate the rapeseed seedling count precisely and to offer a comprehensive study of field-based rapeseed seedling estimation throughout its early growth stages. Moreover, we tried to present a study showing general and user-friendly workflow for executing CNN methods. We also expected that our research offered another perspective in phenotyping and cultivation management for estimating seedling count for crops that have obvious tillering leaves at early growth stages such as soybean and potato. The results based on the particular case were desirable and promising. More detailed data and relevant technical information of our study were deposited to GitHub repository in “LARSC-Lab/Rapeseed_seedling_counting”.

In the future, we will focus on the data accumulation including data from multiple growing seasons and multiple fields in different locations. Moreover, multiple approaches are expected to be employed, and their performances will be compared to identify optimal methods and improve accuracy. A more comprehensive dataset of rapeseed from UAV imagery is expected to be completed and published in the future based on our work, which will promote the research in phenotyping for rapeseed and other crops.

## Conclusion

Utilizing a consumer-grade camera mounted on a UAV for crop phenotyping and vegetation investigation in the field is feasible and efficient. This study attempted to estimate rapeseed stand count in UAV-captured RGB imagery with machine learning. CNN algorithm was used for rapeseed leaf identification and counting. Regression modeling coupled with LOOCV method was used to establish and optimize the relationship between the seedling counting and the number of rapeseed leaves. When the number of CNN-detected rapeseed leaves was brought into seedling counting models, the results demonstrated that our proposed framework performed well and achieved great accuracy. In summary, the following conclusions can be drawn from this study:

(1) The effectiveness of our proposed CNN framework on rapeseed leaf recognition and counting was verified in this study. Overcounting is a common problem during leaf recognition and counting. The overcounting rate was related to the DAP reflecting of the rapeseed growth conditions during leaf development. CNN-recognized rapeseed leaf counting incorporated with overcounting calibration was reliable with an overall *F-score* of more than 90%. On average, 806 out of 812 plants were correctly estimated on 53 DAP at the four- to six- leaf stage. RMSE was nine plants with rRMSE of 2.22% on 53 DAP for the 32-pixel patch size, while the mean RMSE was 12 with mean rRMSE of 2.89% for all patch sizes.
(2) This study demonstrated that DAP influenced the overcounting rate, CNN-recognized leaf results, and seedling counting models. On 46 and 53 DAP, the counting models presented desirable performance. Moreover, a strong correlation (*R*^2^ = 0.835) was also found between coefficients of counting models and DAP. The optimal observation period was on 53 DAP corresponding to the four- to six-leaf stage of rapeseed development.
(3) Based on machine learning approaches, this study proposed a framework for rapeseed stand counting in the field by high-resolution UAV imagery. Our future studies will focus on the collection and evaluation of multiple-year datasets to improve the robustness and reliability of the stand counting models.

## Data Availability Statement

The “Rapeseed_seedling_counting” data that support the findings of this study are available in “LARSC-Lab/Rapeseed_seedling_counting” in GitHub, which can be found at https://github.com/LARSC-Lab/Rapeseed_seedling_counting.

## Author Contributions

JZ, JX, and BZ designed and conducted the remote sensing part of the experiment. QL and GZ designed and conducted the agronomy part of the experiment. JZ, BZ, JX, and CH processed and analyzed the imagery as well as wrote the manuscript. CY guided the study design, advised in data analysis, and revised the manuscript. WY, YS, DZ, CW, TX, and ZJ were involved in the process of the experiment, ground data collection, or manuscript revision. All authors reviewed and approved the final manuscript.

## Conflict of Interest

The authors declare that the research was conducted in the absence of any commercial or financial relationships that could be construed as a potential conflict of interest.

## Footnotes

1 www.fao.org/home/en/

## Abbreviations

CNN: convolutional neural network
DAP: day after planting
GCP: ground control point
GPS: global positioning system
LOOCV: leave-one-out crossing validation
MAE: mean absolute error
RF: random forest
RMSE: root mean square error
rRMSE: relative root mean square error
SVM: support vector machine
UAV: unmanned aerial vehicle
UGV: unmanned ground vehicle.
